# Supplementary figures and images for: EZH2 inhibition decreases neuroblastoma proliferation and in vivo tumor growth
Source: PLoS One. 2021 Mar 9;16(3):e0246244. doi: 10.1371/journal.pone.0246244 (PMC7942994; doi:10.1371/journal.pone.0246244)

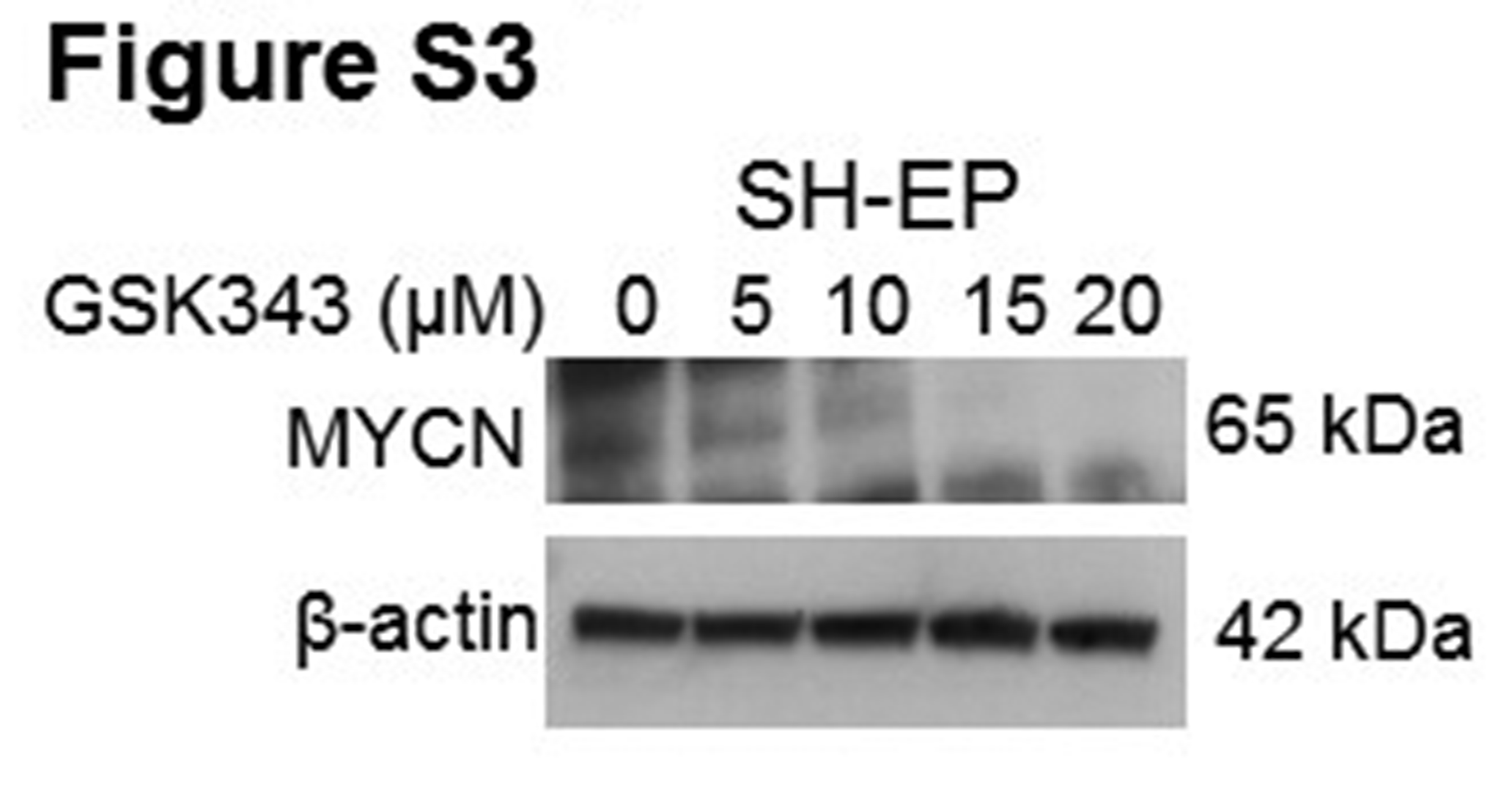

Supplement: S1 Fig — (TIF) [file pone.0246244.s001.tif]

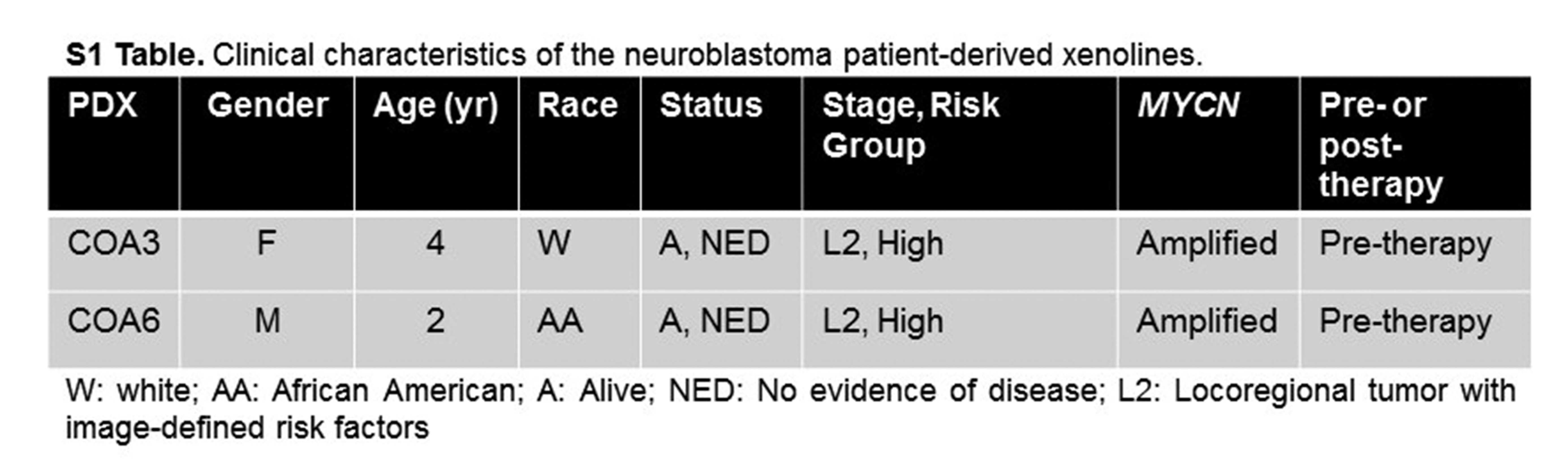

Supplement: S1 Table — (TIF) [file pone.0246244.s002.tif]

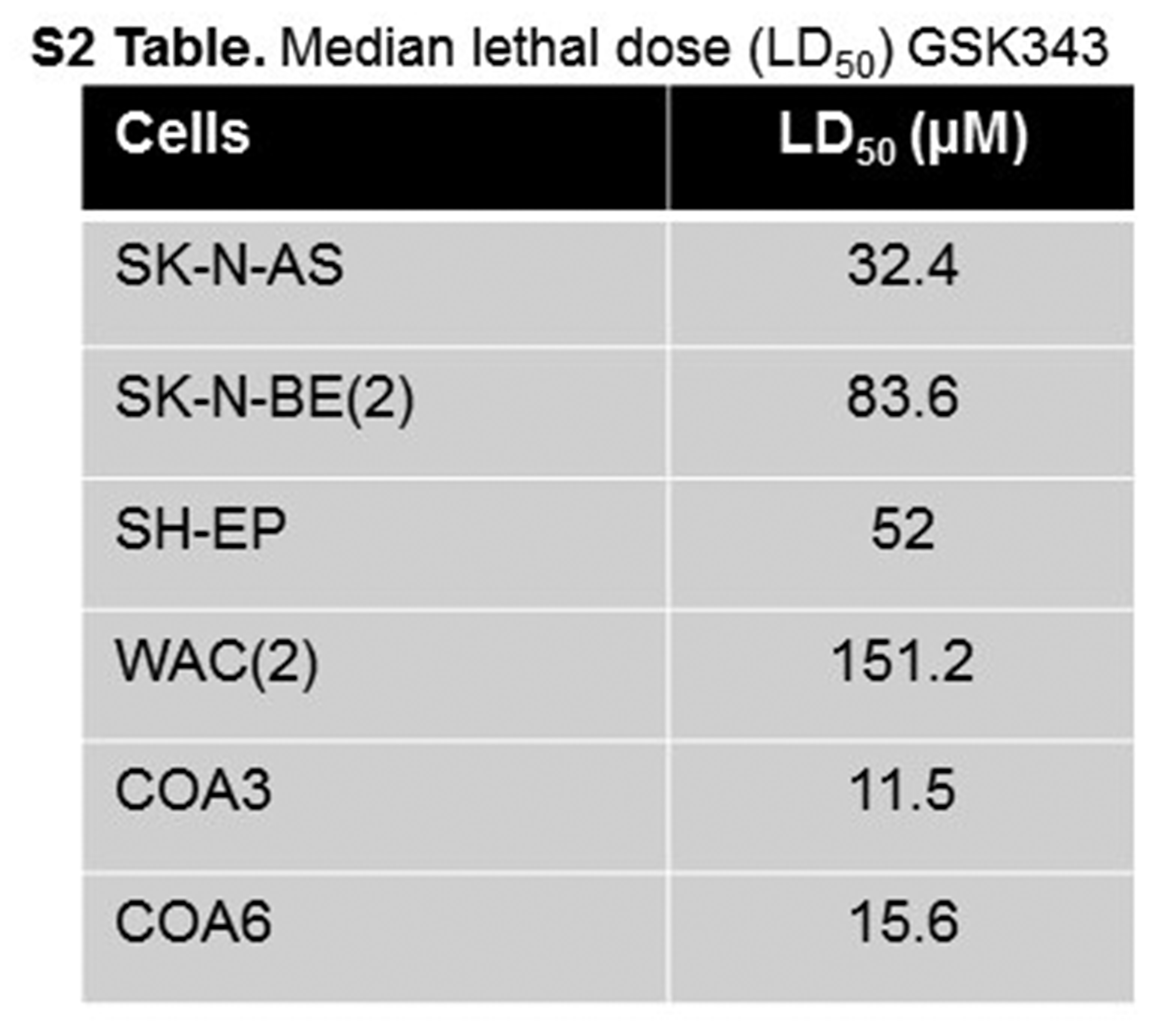

Supplement: S2 Table — (TIF) [file pone.0246244.s003.tif]
