# Supplementary material for: EZH2 inhibition decreases neuroblastoma proliferation and in vivo tumor growth
Source: PLoS One. 2021 Mar 9;16(3):e0246244. doi: 10.1371/journal.pone.0246244 (PMC7942994; doi:10.1371/journal.pone.0246244)
Supplement: S1 Raw images — (PDF) [file pone.0246244.s004.pdf]

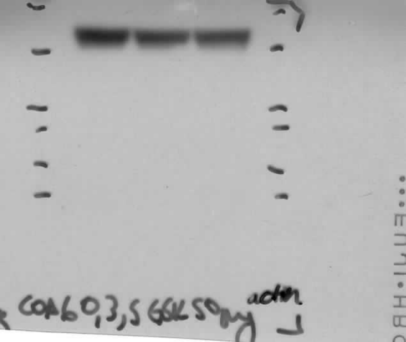
actin

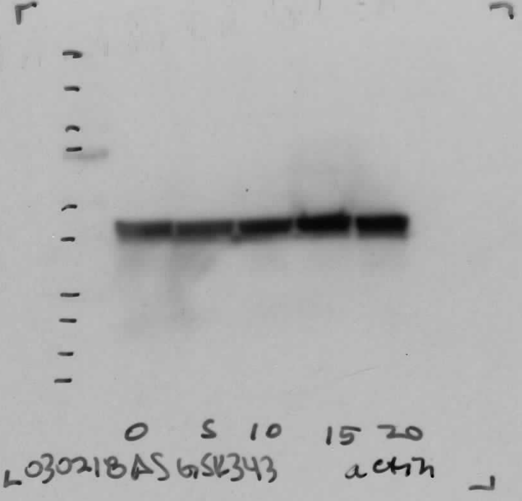
0 5 10 15 20
030218AS GSK343
actin

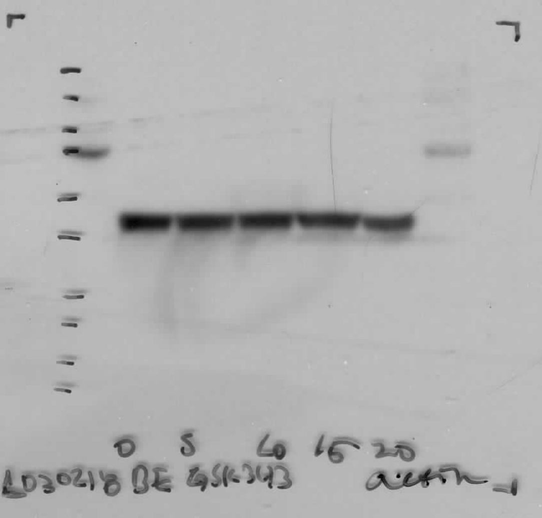
0 5 10 15 20
LD30218 BE GSK343
actin

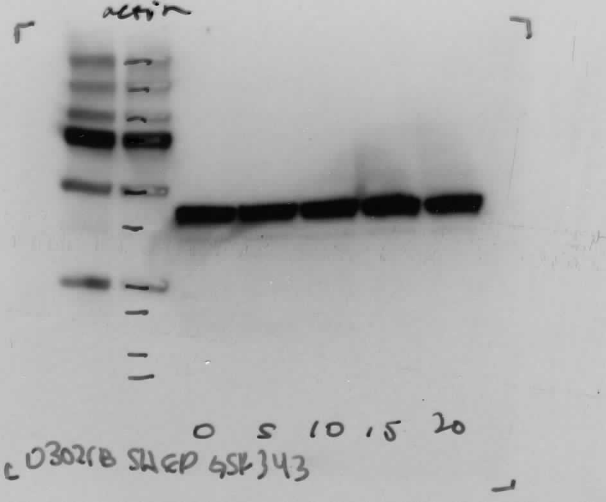
actin
0 5 10 15 20

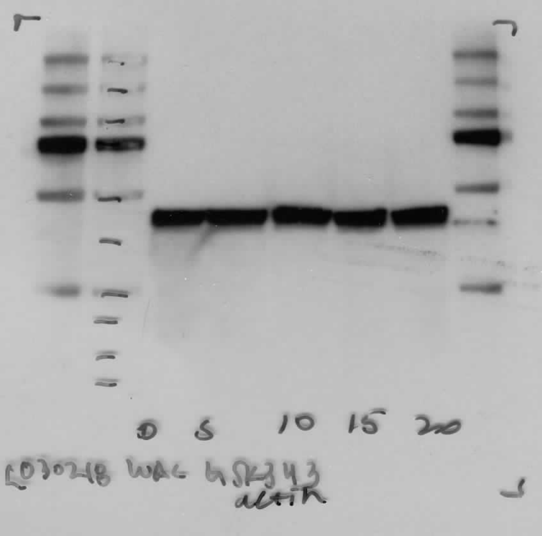
0 5 10 15 20
030218 WAG GSK343
actin

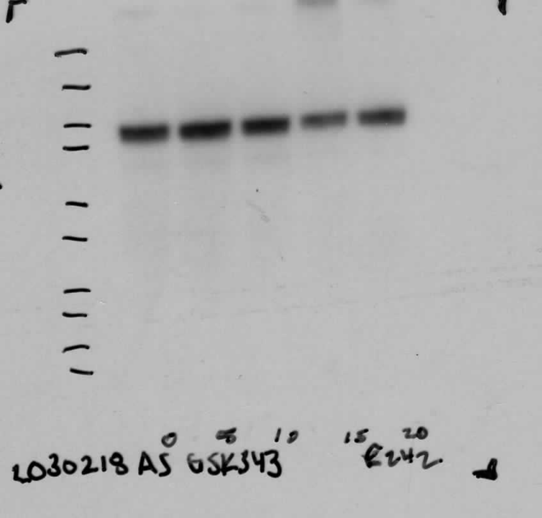
0 5 10 15 20
2030218 AS GSK343 E242.

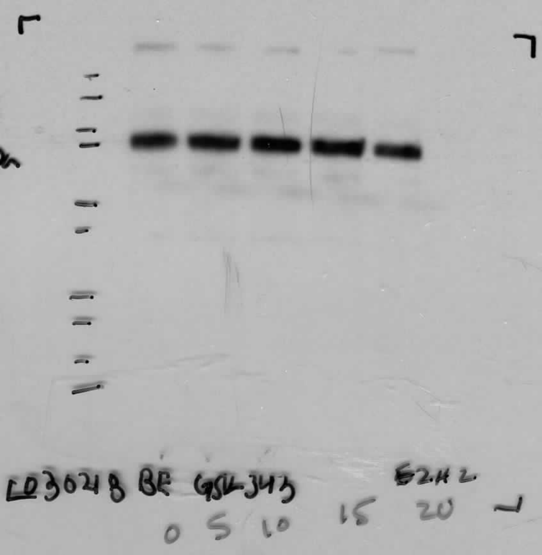
LD30218 BE GSK343
EZH2
0 5 10 15 20

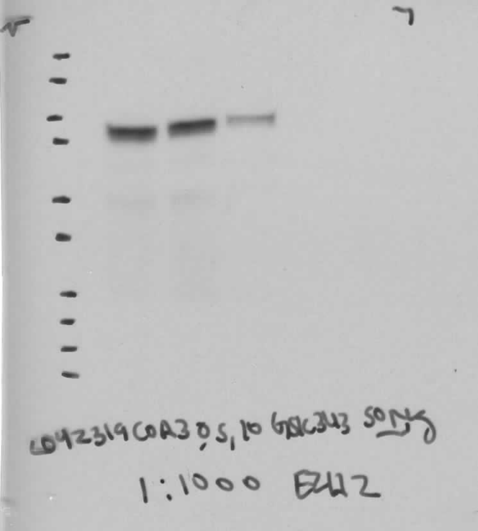
C042319 COA3 0,5,10 GSK343 50µg
1:1000 EZH2

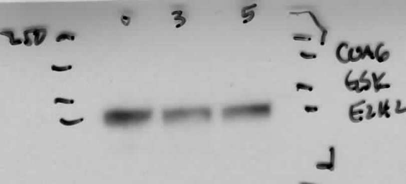
250
3
5
COA6
GSK

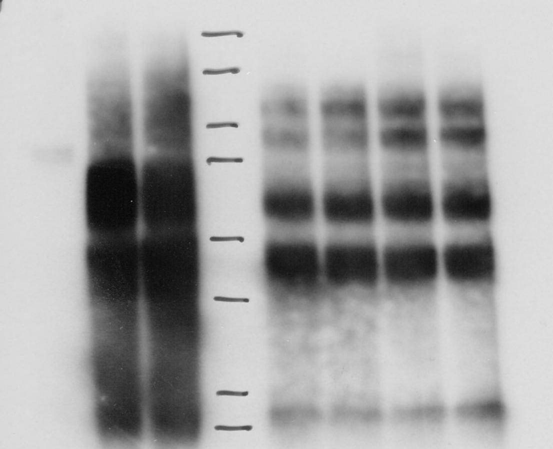

042018 EZH2-FAK COIP

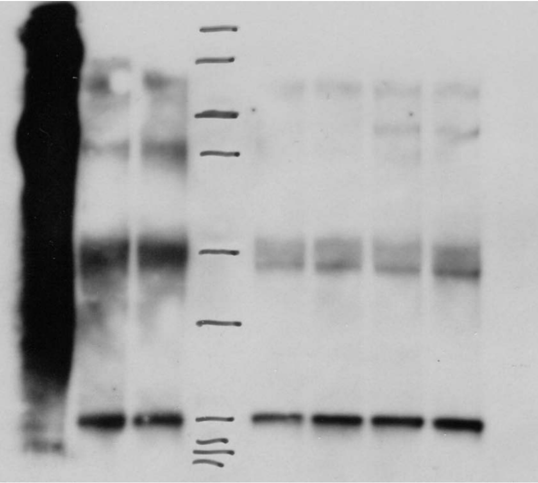

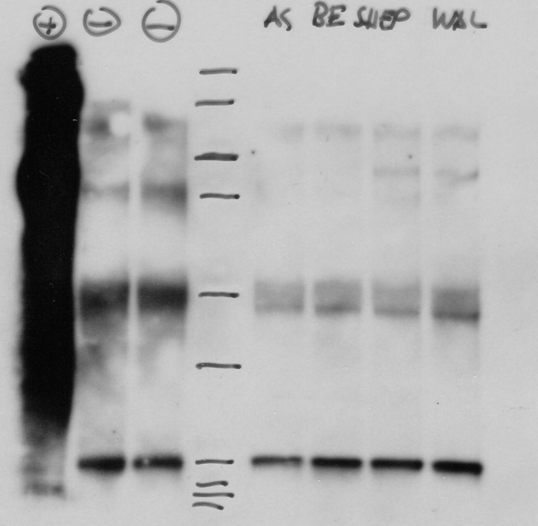

+
−
−
AS
BE
SHEP
WAL

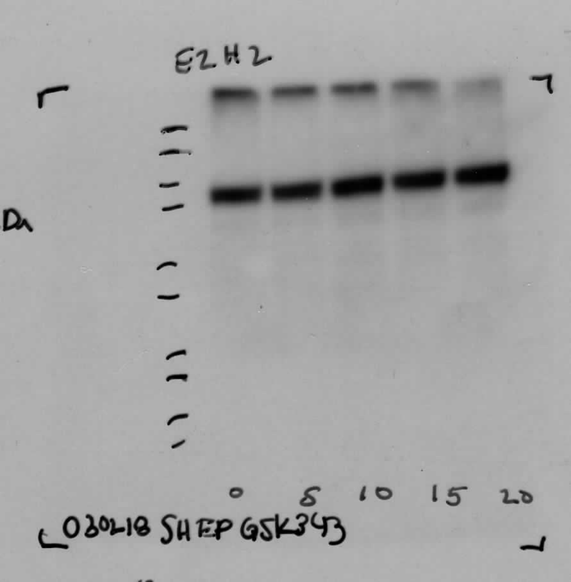
EZH2
0 5 10 15 20
030218 SHEP GSK343

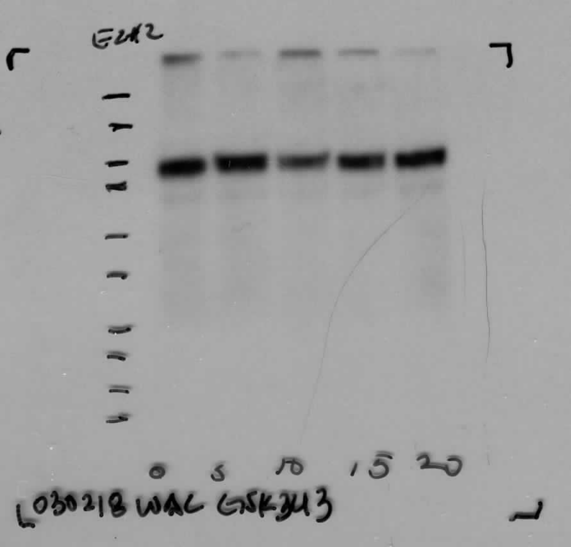
EZH2
0 5 10 15 20
L030218 WAL GSK343

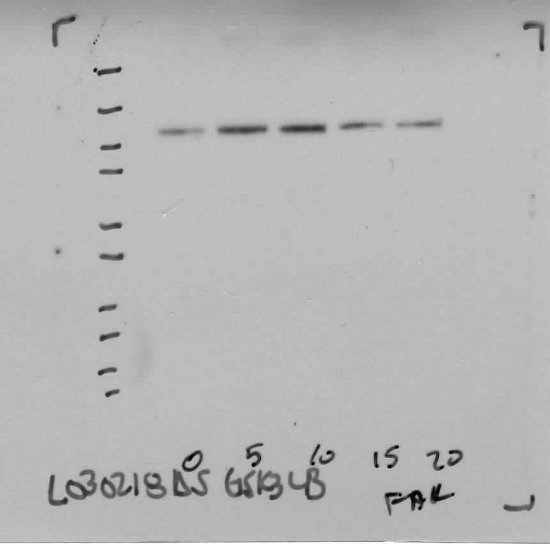
0 5 10 15 20
L030218DS G5B3 4B
FAK

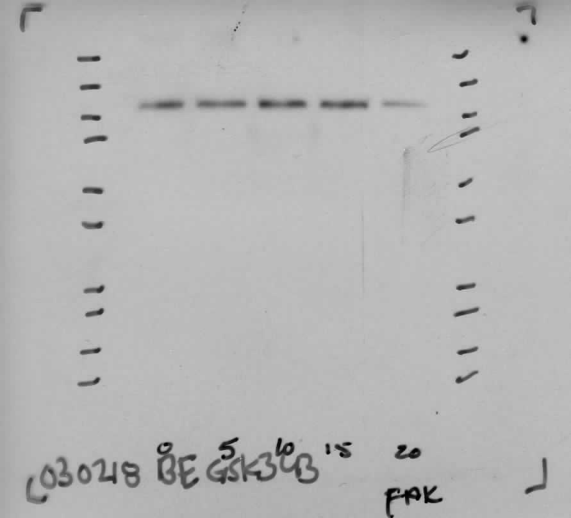

0
5
10
15
20
030218 BE GSK3 CB
FAK

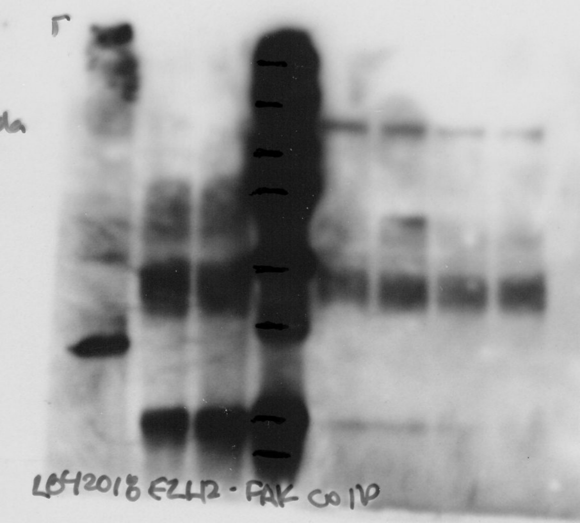
L6/42018 EZH2 - FAK co IP

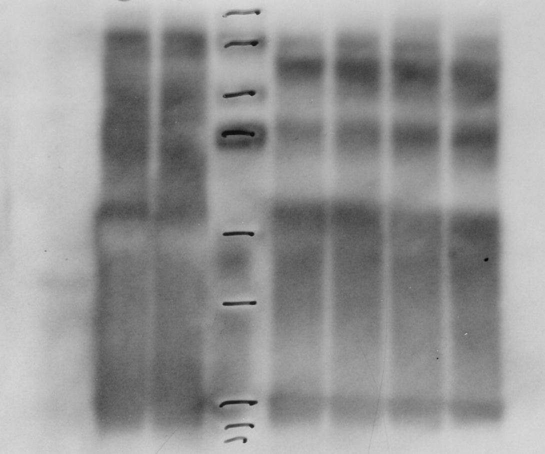

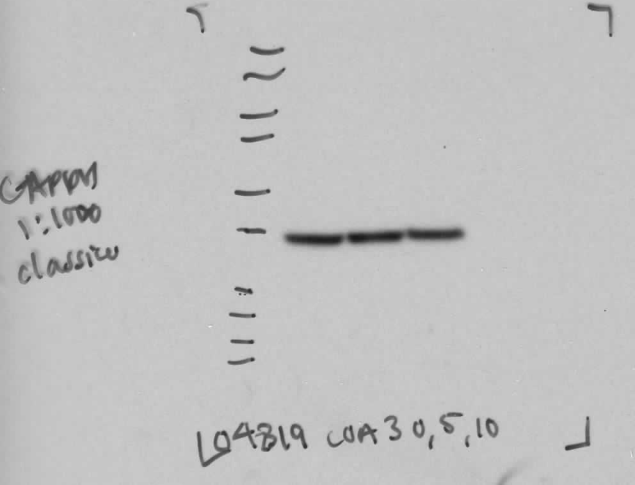
GAPDH
1:1000
classico
L 04819 COA3 0,5,10

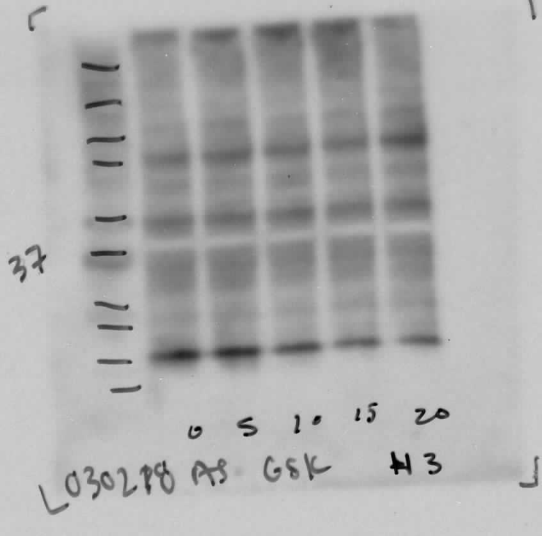
37
0 5 10 15 20
L030288 AS GSK H3

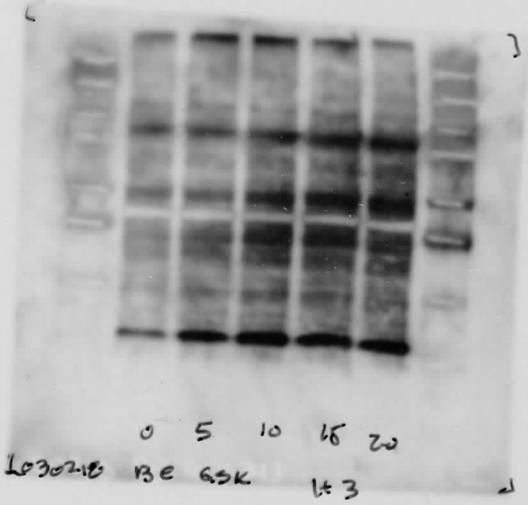
0 5 10 15 20
L030218 BE GSK
Lt 3

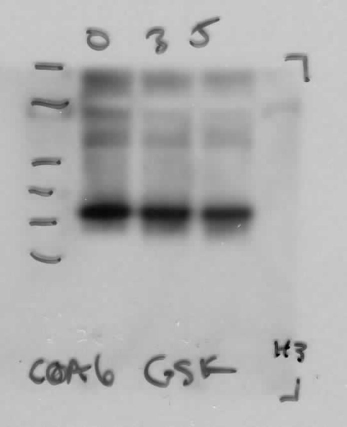
0 3 5
COA6 GSK
H3

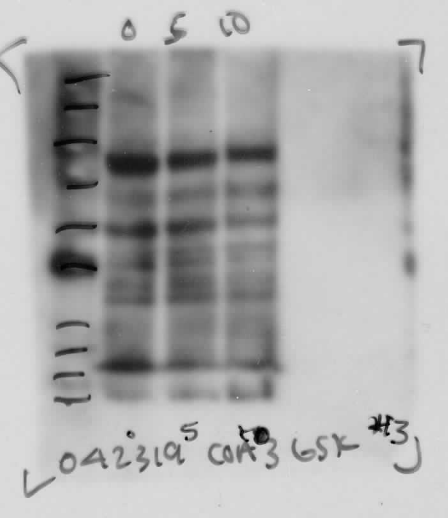
0 5 10
042319 COA3 GSK #3

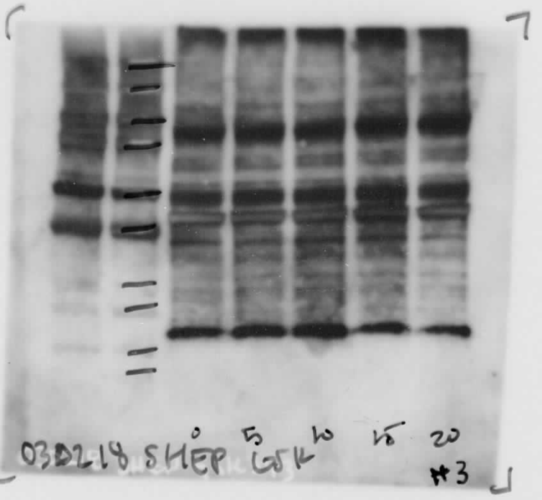
0
5
10
15
20
030218 SHEP GSK
#3

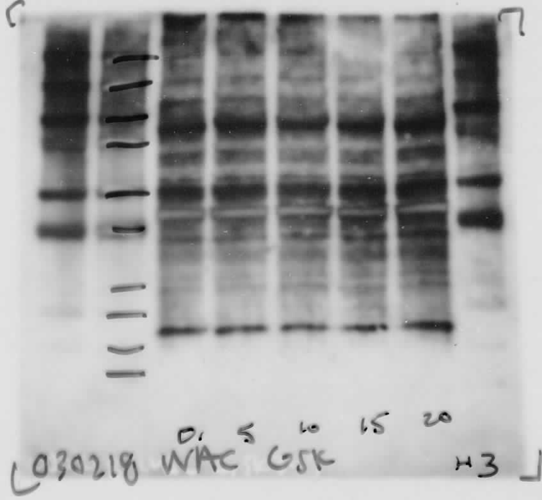
0 5 10 15 20
030218 WAC G5K
H3

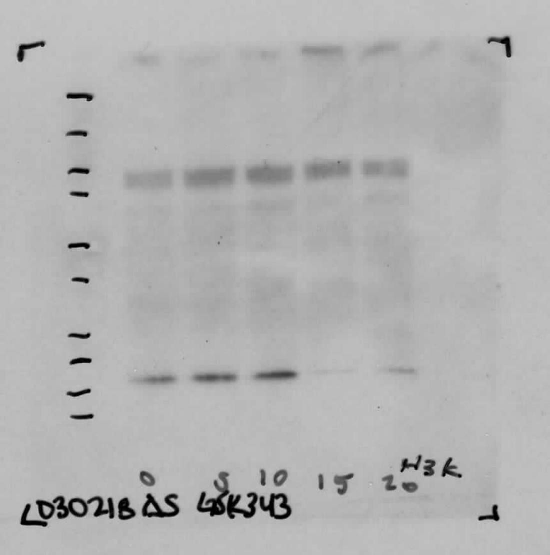

H3K
0 5 10 15 20
L030218 ΔS GSK343

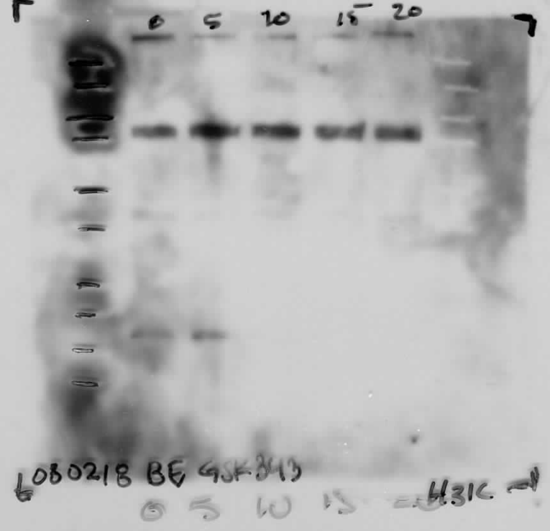
0 5 10 15 20
080218 BE
0 5 10 15

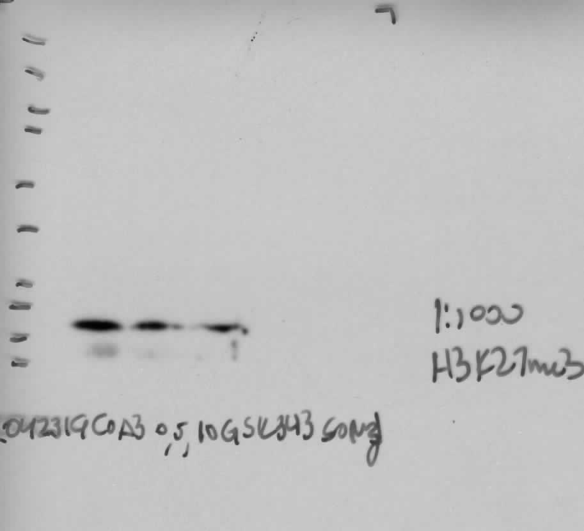
1:1000
H3K27me3
0412319COA3 0,5,10 GSK343 50ug

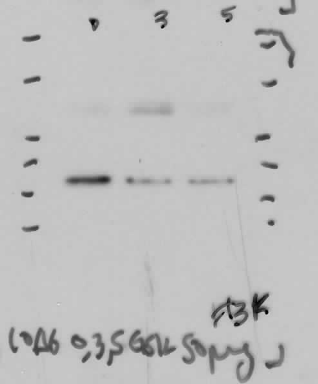
0
3
5
13K
LoΔ6 0,3,5 G6K 50μg

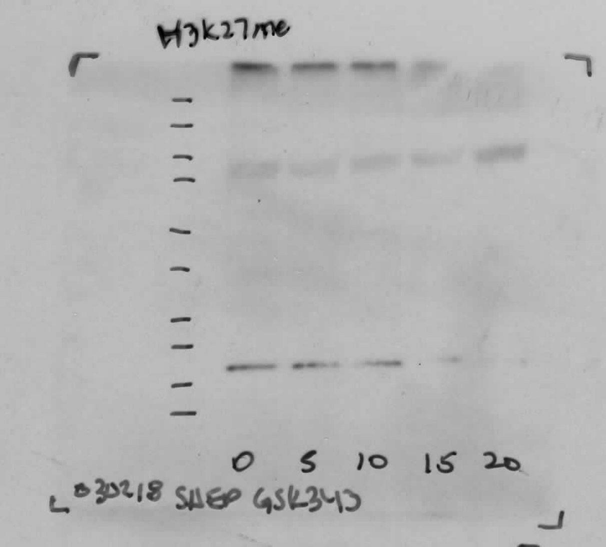
H3K27me
0 5 10 15 20
030218 SHEP GSK343

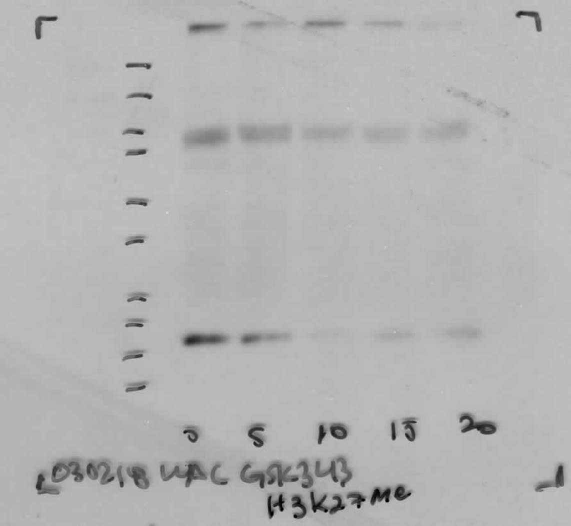
0 5 10 15 20
030218 WAC GSK343
H3K27Me

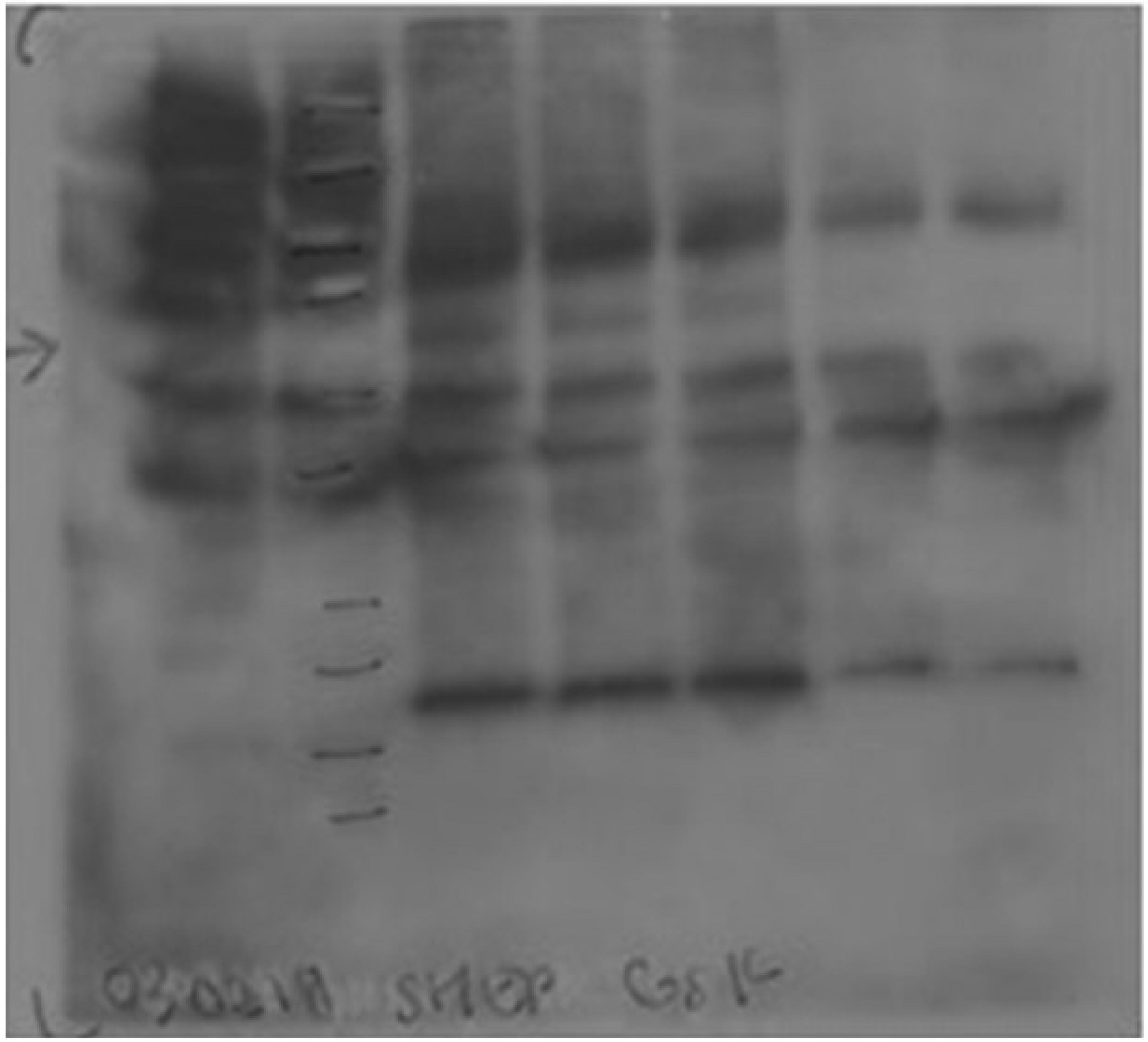

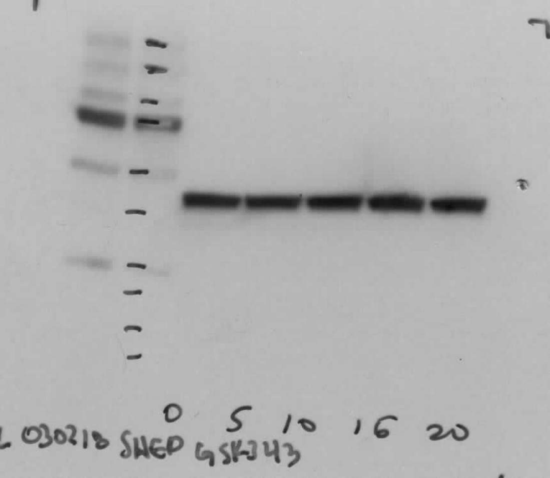
0 5 10 16 20
030218 SHEP GSK343

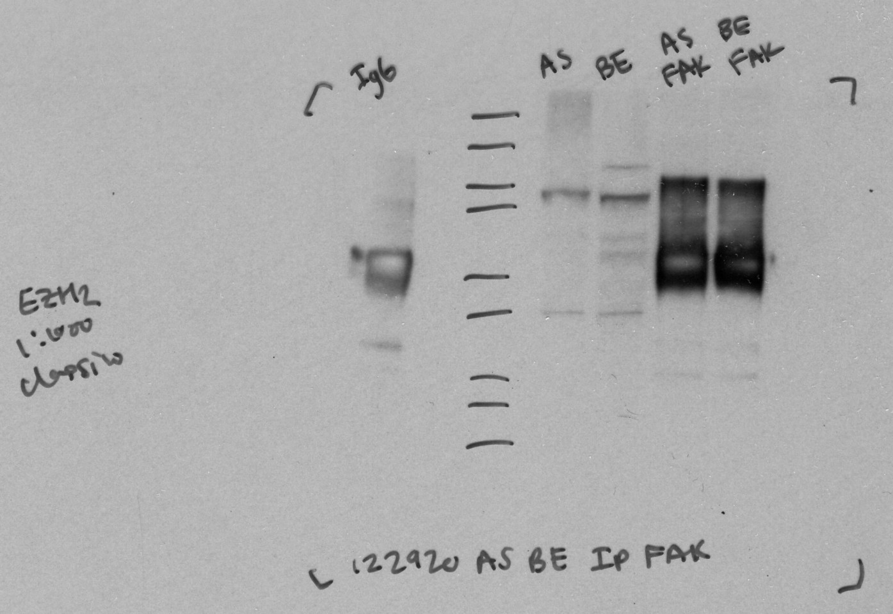
IgG
AS
BE
AS FAK
BE FAK
EZH2
1:1000
122920 AS BE IP FAK

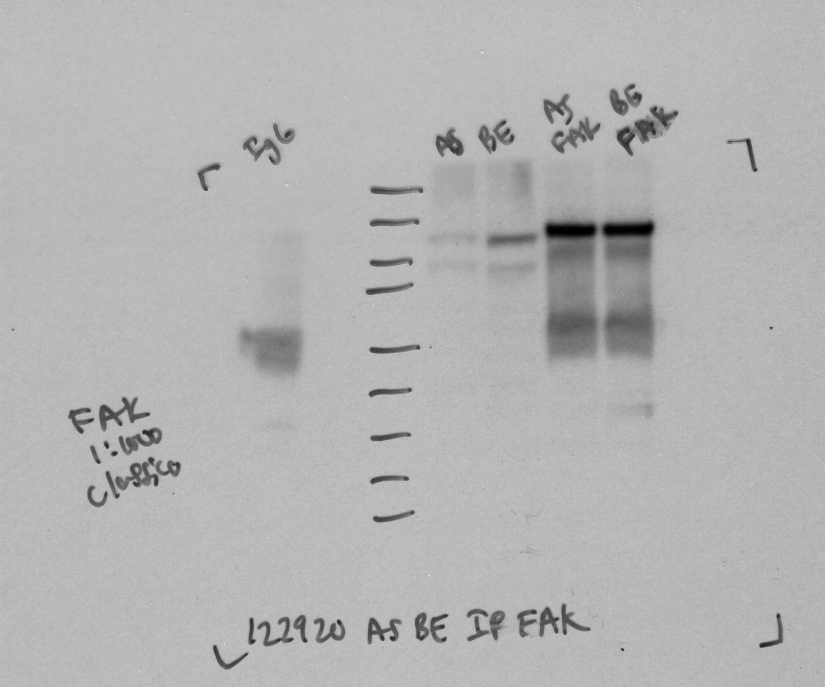
IgG
AS
BE
AS FAK
BE FAK
FAK 1:1000 clasico
122920 AS BE IP FAK

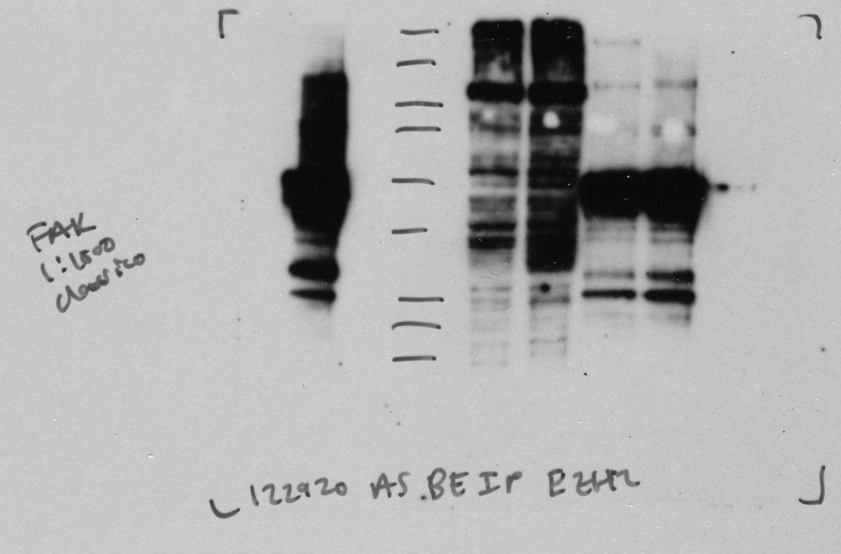
FAK
1:1500
122920 AS BE IP E2H2

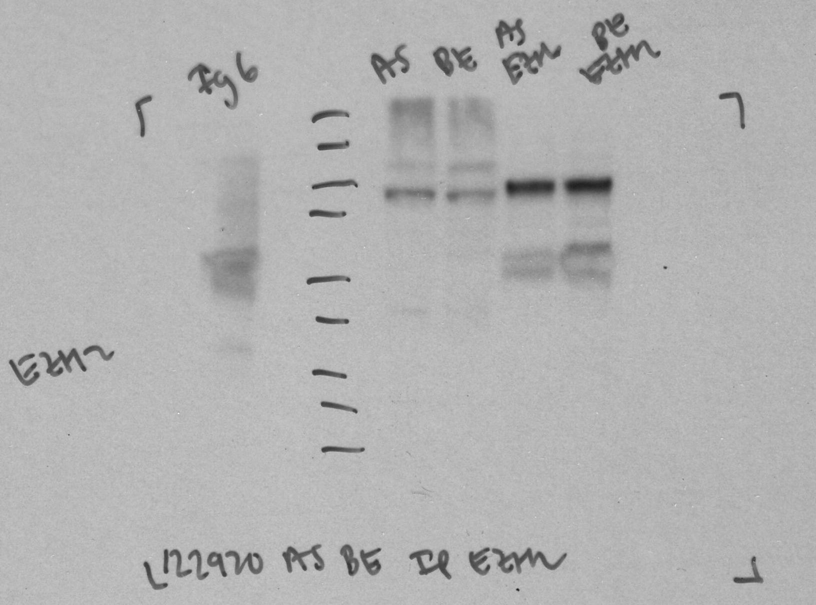
IgG
AS
BE
AS EZH
BE EZH
EZH2
122920 AS BE IP EZH
