# Supplementary material for: EZH2 inhibition decreases neuroblastoma proliferation and in vivo tumor growth
Source: PLoS One. 2021 Mar 9;16(3):e0246244. doi: 10.1371/journal.pone.0246244 (PMC7942994; doi:10.1371/journal.pone.0246244)
Supplement: Attachment — Submitted filename: Reviewers queries.docx [file pone.0246244.s006.docx]

The revised manuscript is much improved. There remain a few issues noted below that I feel are significant enough to require attention. Specifically reviewer #2 points out an issue with the conclusion of Figure 1 and Reviewer #2 notes that the data supporting interaction of EZH2 and FAK remains poor in Figure 6D/E and some discussion is required for Figure 7. I also would expect to see the lysates run on the same gels and ideally a much improved signal to noise ratio. I believe these are generally straightforward requests and will better support the conclusions. 

*Thank you for your comments and suggestions. We have revised the description of Figure 1 based upon the suggestions of Reviewer #1. We have also performed additional experiments again showing an interaction between FAK and EZH2. These IP Westerns have been provided in revised Figure 6. We believe that they are of better quality. We have also provided a detailed explanation for the queries posed by Reviewer #2.*

 Reviewers' comments:

Reviewer's Responses to Questions

**Comments to the Author**

1. If the authors have adequately addressed your comments raised in a previous round of review and you feel that this manuscript is now acceptable for publication, you may indicate that here to bypass the “Comments to the Author” section, enter your conflict of interest statement in the “Confidential to Editor” section, and submit your "Accept" recommendation.

Reviewer #1: All comments have been addressed

Reviewer #2: (No Response)

Reviewer #3: (No Response)

2. Is the manuscript technically sound, and do the data support the conclusions?

The manuscript must describe a technically sound piece of scientific research with data that supports the conclusions. Experiments must have been conducted rigorously, with appropriate controls, replication, and sample sizes. The conclusions must be drawn appropriately based on the data presented.

 Reviewer #1: Yes

Reviewer #2: Yes

Reviewer #3: Partly

*See revisions*

3. Has the statistical analysis been performed appropriately and rigorously?

 Reviewer #1: Yes

Reviewer #2: Yes

Reviewer #3: Yes

4. Have the authors made all data underlying the findings in their manuscript fully available?

The [PLOS Data policy](https://protect-us.mimecast.com/s/3wf1C4xJjqsB81DxhBtunv?domain=plosone.org) requires authors to make all data underlying the findings described in their manuscript fully available without restriction, with rare exception (please refer to the Data Availability Statement in the manuscript PDF file). The data should be provided as part of the manuscript or its supporting information, or deposited to a public repository. For example, in addition to summary statistics, the data points behind means, medians and variance measures should be available. If there are restrictions on publicly sharing data—e.g. participant privacy or use of data from a third party—those must be specified.

Reviewer #1: Yes

Reviewer #2: Yes

Reviewer #3: Yes

5. Is the manuscript presented in an intelligible fashion and written in standard English?

PLOS ONE does not copyedit accepted manuscripts, so the language in submitted articles must be clear, correct, and unambiguous. Any typographical or grammatical errors should be corrected at revision, so please note any specific errors here.

Reviewer #1: Yes

Reviewer #2: Yes

Reviewer #3: Yes

6. Review Comments to the Author

Please use the space provided to explain your answers to the questions above. You may also include additional comments for the author, including concerns about dual publication, research ethics, or publication ethics. (Please upload your review as an attachment if it exceeds 20,000 characters)

**Reviewer #1**: All of my comments and questions have been addressed

*Thank you.*

**Reviewer #2**: Overall, the authors have carefully addressed the reviewers’ comments and the manuscript is ready for publication after one minor revision of the text. The interpretation of data in Figure 1 needs clarification (lines 318-327). The title of Figure 1 is “EZH2 inhibitor, GSK343, decreased expression of H3K27me3.” This is not correct for the authors now show that H3 expression was not changed in the presence of GSK343. Based on inhibitor mechanism, the EZH2 inhibitor decreased the methylation of Histone 3 at Lysine 27. This should be corrected in the title for Figure 1, the legend title for Figure 1, results section for Figure 1 as well as in the discussion text for Figure 1 (lines 497- 499).

New title suggestion: EZH2 inhibitor, GSK343, decreased tri-methylation of Histone 3 at Lysine 27.

*Thank you. We made these changes to the Results section, the Legend title for Figure 1 and the text for Figure 1 legend. We have also made the changes to the discussion text lines 501-503.*

**Reviewer #3**: In the revised version, authors modified several parts of the paper appropriately, however, my comments 6 and 7 are still unresolved.

1. Fig.1, as a loading control, total histone H3 WB is better.
＞Thank you. H3 has been added to the immunoblots and to the methods section.
The time of incubation with GSK343 should be mentioned in the legend.
＞The time of incubation for Fig. 1 was 24 hours has been added to the results section and the legend.

I accept their answer.

*Thank you.*


2. Fig.2e: How authors can argue that GSK343 treated cells (right panel) demonstrated significant reduction in ability to heal the scratch compared to untreated cells (left panel)?
Authors should try to quantify the reduction and to present the statistical significance.
>The photomicrograph in Fig. 2 G was presented as a representative example of the scratch assay plates. The scratch assays were completed in triplicate with at least three biologic replicates. The area of the scratch that was open was quantified with a computer program and reported as fold change comparing areal open after 24 hours to the area open at 0 hours, immediately following the wounding of the plate.

I accept their answer.
*Thank you.*

3. Fig.3: The representative xenograft photos should be presented for readers.
We deeply regret that we do not have any representative xenograft photos. They were not obtained at the time of animal euthanasia.

I accept their answer.

*Thank you.*

4. Fig.4: Authors indicated that GSK343 significantly decreased the proliferation and viability of the NB cells.
Although they argued that migration and invasion were down-regulated by GSK343, I think their experiments did not address the migration and invasion because of difference of viable cell number by GSK343 treatments.
>In Figure 2 and Figure 4, the concentrations of GSK343 chosen for the motility studies was well below the calculated LD50 of the drug (Supplemental Table 2). Although GSK343 had some effect on viability at lower concentrations, we believe that most of the effect on the motility phenotype was not attributed to decreased cell death, but from the effects of the drug, since these effects were noted at lower concentrations. The same was seen for proliferation. Proliferation was significantly decreased at concentrations of GSK343 that were below the LD50 concentrations.

I accept their answer.
*Thank you.*

5. Fig.6B and C: EZH2 mainly locates in nucleus and FAK (PTK2 may be better) mainly locates cytoplasm and nucleus. I can’t distinguish nucleus in Fig.6B because authors did not indicate the single DAPI staining photo. Further, the quality of IHC was not good.
>We have provided new IHC pictures with DAPI added for Figure 6 B and the figure legend has been revised. The nucleus is represented by the blue DAPI staining.

I accept their modification.

*Thank you.*

6. Fig.6D and E: The quality of IP-WB experiments were not good because background signals were high. I think direct WB results by using total cell lysates were required for these experiments and MW marker lanes also should be indicated. Further, location of the Ig bands should be indicated in these IP-WBs.
>We agree that the background signal was high on the IP Westerns. Unfortunately, since these studies required an inordinate amount of protein (500 µg) to be loaded, it led to significant background. However, we believe that these blots are important for the study since they demonstrate an interaction between the two proteins, FAK and EZH2 that has not been demonstrated previously in neuroblastoma. These blots also lend credence to the concept that alterations in FAK are responsible for some of the phenotypic changes seen following treatment with GSK343.

I agree that interaction between EZH2 and FAK is important for their work. However, the present IP-WB results has too low quality to confirm that. I do require direct WB results by using total cell lysates in the same gel experiments and reduction of the background. Further, IgG lane should not be cut form the sample lanes. My suggestion is anti-EZH2 ab from Millipore may be better than the ab they used for WB. If they can not improve their endogenous IP-WBs, they need to think about detection of the interaction by transfection of one of the molecules in NB cells.

*Thank you for your comments and for your appreciation of the difficulties in obtaining good quality IP-WBs. We have repeated the IP Western experiments and have provided these new immunoblots in the revised manuscript in Figure 6 D, E. They again support an interaction between FAK and EZH2. We also feel that they are of excellent quality. The previous blots remain in supplemental data for reference.*

7. R2 database analysis using Kocak database indicated that PTK2 low expression significantly related to the worse prognosis of NB patients. Authors need to discuss the discrepancy.
>We have found in our laboratory that increased FAK activation and expression was associated with worse prognosis and with amplification of MYCN in neuroblastoma [12, 13].

Ref 12 is the following: Augmented MYCN Expression Advances the Malignant Phenotype of Human Neuroblastoma Cells: Evidence for Induction of Autocrine Growth Factor Activity? I could not find the K-M analysis in that. Further, not only KOCAK DB (n=649) but also SEQC DB (n=498) indicated that PTK2 low expression significantly related to the worse prognosis of NB patients. Authors need to discuss the discrepancy in the revised version.

*Thank you for your thorough investigation of the Kocak (R2) and other gene databases. We agree that a discrepancy exists between the gene expression data and the data that address the protein expression and phosphorylation status of the FAK protein in neuroblastoma. The Kocak (R2) and other databases, that examined gene expression, revealed that lower FAK (PTK2) gene expression is associated with worse overall survival in neuroblastoma. However, the studies examining FAK protein expression have indicated that higher protein expression was associated with worse disease including MYCN amplification and metastasis [1-3]. We have a few explanations for these discrepancies. First, gene expression does not always translate and equate with protein expression due to translational and post-translational modifications of gene products. Second, most of the protein data in the literature indicate a relation between high FAK expression and patients with high-risk disease or amplification of the MYCN oncogene, but these studies focused on MYCN amplified or high-risk disease and often did not include patients with low or intermediate risk tumors. Since the gene expression datasets include all comers for the disease including very low, low and intermediate risk patients, there may be factors related to disease stratification that may contribute to the conflicting findings. In fact, in one of our earliest publications investigating FAK protein expression with immunohistochemistry in 70 patient samples, patient overall or event free survival did not relate to FAK staining in a statistically significant manner. Positive IHC staining for FAK was, however, associated with MYCN amplified disease in high-risk patients [3]. We have added this discussion to the discussion section lines 611-627 of the revised manuscript.*

8. To study the effects of GSK343 on the FAK (PTK2) target molecules and pathways in NB cells will provide the important information for their study.
>We agree that these will be important areas to pursue in future studies and are the subject of our ongoing studies.

I accept the argument.

*Thank you.*

 References for revisions

1. Kratimenos P, Koutroulis I, Syriopoulou V, Michailidi C, Delivoria-Papadopoulos M, Klijanienko J, Theocharis S. FAK-Src-paxillin system expression and disease outcome in human neuroblastoma. Pediatr Hematol Oncol. 2017 May;34(4):221-230. doi: 10.1080/08880018.2017.1360969. Epub 2017 Oct 17. PMID: 29040002
2. Lee S, Qiao J, Paul P, O'Connor KL, Evers MB, Chung DH. FAK is a critical regulator of neuroblastoma liver metastasis. Oncotarget. 2012 Dec;3(12):1576-87. doi: 10.18632/oncotarget.732. PMID: 23211542
3. Beierle EA, Massoll NA, Hartwich J, Kurenova EV, Golubovskaya VM, Cance WG, McGrady P, London WB. Focal adhesion kinase expression in human neuroblastoma: immunohistochemical and real-time PCR analyses. Clin Cancer Res. 2008 Jun 1;14(11):3299-305. doi: 10.1158/1078-0432.CCR-07-1511. PMID: 18519756
